# Evolution of an Artificial Intelligence-Powered Application for Mammography

**DOI:** 10.3390/diagnostics15070822

**Published:** 2025-03-24

**Authors:** Yuriy Vasilev, Denis Rumyantsev, Anton Vladzymyrskyy, Olga Omelyanskaya, Lev Pestrenin, Igor Shulkin, Evgeniy Nikitin, Artem Kapninskiy, Kirill Arzamasov

**Affiliations:** 1 Research and Practical Clinical Center for Diagnostics and Telemedicine Technologies of the Moscow Health Care Department, 127051 Moscow, Russia; vasilevya1@zdrav.mos.ru (Y.V.); vladzimirskijav@zdrav.mos.ru (A.V.); omelyanskayaov@zdrav.mos.ru (O.O.); pestreninld@zdrav.mos.ru (L.P.); shulkinim@zdrav.mos.ru (I.S.); arzamasovkm@zdrav.mos.ru (K.A.); 2 Department of Information Technology and Medical Data Processing, I.M. Sechenov First Moscow State Medical University (Sechenov University), 119991 Moscow, Russia; 3 Celsus (Medical Screening Systems), Viktorenko St., Bldg. 11, Room 21N, 125167 Moscow, Russia; e.nikitin@celsus.ai (E.N.); a.kapninskiy@celsus.ai (A.K.); 4 Department of Artificial Intelligence Technologies, MIREA—Russian Technological University, 119454 Moscow, Russia

**Keywords:** artificial intelligence, radiology, mammography, software, software validation

## Abstract

**Background:** The implementation of radiological artificial intelligence (AI) solutions remains challenging due to limitations in existing testing methodologies. This study assesses the efficacy of a comprehensive methodology for performance testing and monitoring of commercial-grade mammographic AI models. **Methods:** We utilized a combination of retrospective and prospective multicenter approaches to evaluate a neural network based on the Faster R-CNN architecture with a ResNet-50 backbone, trained on a dataset of 3641 mammograms. The methodology encompassed functional and calibration testing, coupled with routine technical and clinical monitoring. Feedback from testers and radiologists was relayed to the developers, who made updates to the AI model. The test dataset comprised 112 medical organizations, representing 10 manufacturers of mammography equipment and encompassing 593,365 studies. The evaluation metrics included the area under the curve (AUC), accuracy, sensitivity, specificity, technical defects, and clinical assessment scores. **Results:** The results demonstrated significant enhancement in the AI model’s performance through collaborative efforts among developers, testers, and radiologists. Notable improvements included functionality, diagnostic accuracy, and technical stability. Specifically, the AUC rose by 24.7% (from 0.73 to 0.91), the accuracy improved by 15.6% (from 0.77 to 0.89), sensitivity grew by 37.1% (from 0.62 to 0.85), and specificity increased by 10.7% (from 0.84 to 0.93). The average proportion of technical defects declined from 9.0% to 1.0%, while the clinical assessment score improved from 63.4 to 72.0. Following 2 years and 9 months of testing, the AI solution was integrated into the compulsory health insurance system. **Conclusions:** The multi-stage, lifecycle-based testing methodology demonstrated substantial potential in software enhancement and integration into clinical practice. Key elements of this methodology include robust functional and diagnostic requirements, continuous testing and updates, systematic feedback collection from testers and radiologists, and prospective monitoring.

## 1. Introduction

The early detection of breast cancer is essential to improving patient outcomes, and mammography stands at the forefront of breast cancer screening and diagnosis. Mammography, as a widely adopted imaging technique, has demonstrated efficacy in identifying breast abnormalities before progression to advanced stages [1,2,3,4]. Studies have shown that early detection significantly increases treatment options and improves survival rates [5,6,7,8]. Thus, mammography plays a critical role in public health efforts aimed at reducing breast cancer morbidity and mortality.

In recent years, the integration of artificial intelligence (AI) and machine learning technologies into healthcare has gained considerable traction, particularly within the field of radiology. These innovations improve diagnostic accuracy and efficiency, transforming the landscape of medical imaging [9,10,11,12]. By leveraging vast datasets and complex algorithms, AI systems support radiologists’ decision-making, enabling more nuanced and precise interpretations of imaging results [13,14]. The incorporation of AI into radiological practice streamlines workflows and allows healthcare professionals to focus on patient care [15,16].

Mammography represents one of the most extensively researched applications of AI. AI solutions demonstrate substantial promise as diagnostic support tools, exhibiting remarkable speed and accuracy in mammogram analysis [17,18,19,20]. AI assists radiologists by improving detection rates, reducing false-positive and false-negative results, and achieving better diagnostic outcomes [21,22]. AI addresses several challenges currently faced by radiologists, including an increasing workload from rising screening volumes, demand for high throughput in clinical environments, and the need for continuous training in an evolving healthcare landscape [23,24,25].

Despite these advantages, the integration of mammographic AI remains a challenge. One major issue is the need for external validation of AI models to confirm their effectiveness across various populations and clinical scenarios [26]. External validation involves testing AI models with data acquired from sources different from those used during the development to ensure that the algorithm’s applicability extends beyond its training data [27]. However, most studies of AI in radiology are limited to a single institution and lack external validation [27,28,29]. Although some researchers are expanding their datasets, such single-center studies fail to accurately reflect real-world environments. Additionally, most studies employ a retrospective design, which prevents an assessment of AI generalizability, potentially undermining its integration into clinical decision-making [30,31,32,33,34].

Furthermore, a multicenter and prospective study design alone may not be sufficient for the successful deployment of AI into clinical practice. First, integration of radiological AI requires radiologist feedback during the testing and utilization to refine AI performance and usability. It is essential that AI solutions be designed with the end user in mind [10,35,36]. Secondly, iterative development and regular updates help AI to stay relevant and effective. An update mechanism is necessary to continually refine AI models to keep up with the needs of clinical practice [14,37,38,39,40]. However, both radiologist feedback and continuous AI updating are still missing in most studies. The scientific and regulatory fields rely on the “build and freeze” framework, requiring developers to validate their model on static datasets, report the results, and then apply for regulatory approval [41].

Routine testing and monitoring of AI models enable the identification of weaknesses and provide developers with insights supporting improvement [42,43,44]. Several publications provide methodologies for testing and monitoring radiological AI models [45,46,47,48]. These methodologies are universal across AI fields including medicine and radiology and do not take into account the specifics of imaging modalities, such as mammography. The cited papers give instructions for use and do not contain original findings and use case scenarios. Finally, they lack the concept of continuous learning, which is essential for AI model performance in routine clinical scenarios.

This paper evaluates the effectiveness of a multi-stage testing and monitoring methodology applied to a mammographic AI model for enhancing its performance and reliability. This paper targets AI developers, radiologists, and clinical researchers, offering valuable insights that can contribute to enhancing the practical application of AI in breast cancer screening and diagnosis while ensuring the highest standards of patient care.

## 2. Materials and Methods

### 2.1. Study Setting

This study was conducted as part of the Moscow Computer Vision Experiment (hereafter referred to as “the Experiment”). The goal of the Experiment is to investigate the integration of AI technologies into radiological practice. Initiated in 2020 and ongoing until the present (2025), the Moscow Center for Diagnostics & Telemedicine assesses and compares AI solutions from various vendors to identify and integrate the best-performing models into Moscow’s unified picture archiving and communication system (PACS). Over the course of the Experiment, 52 unique AI models have processed 13,925,132 imaging studies [49].

### 2.2. AI Solution Overview

**Figure 1:**
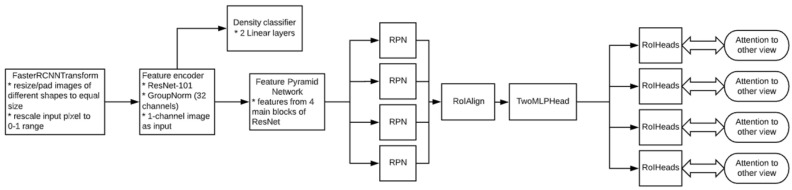
Model architecture. * Module components or actions performed.

This paper investigates the Celsus Mammography^®^ AI model [50]. The model’s primary task is to detect and classify signs of breast cancer on mammograms. For each breast, the model generates a prediction score ranging from 0 to 1, with 1 indicating the highest probability of breast cancer. The neural network is based on the Faster-RCNN architecture [51] with a ResNet-50 backbone module [52] (Figure 1). In 2021, the AI solution received a market authorization certificate from Russia’s National Regulatory Authority, Roszdravnadzor [53], and a Conformité Européenne (CE) mark [54]. These certifications confirm the device’s compliance with safety standards for medical use. To enhance this study’s reliability, we collaborated with the developer to obtain detailed insights into the AI architecture and employed datasets, as outlined in Supplementary Data S1.

### 2.3. Study Design

**Figure 2:**
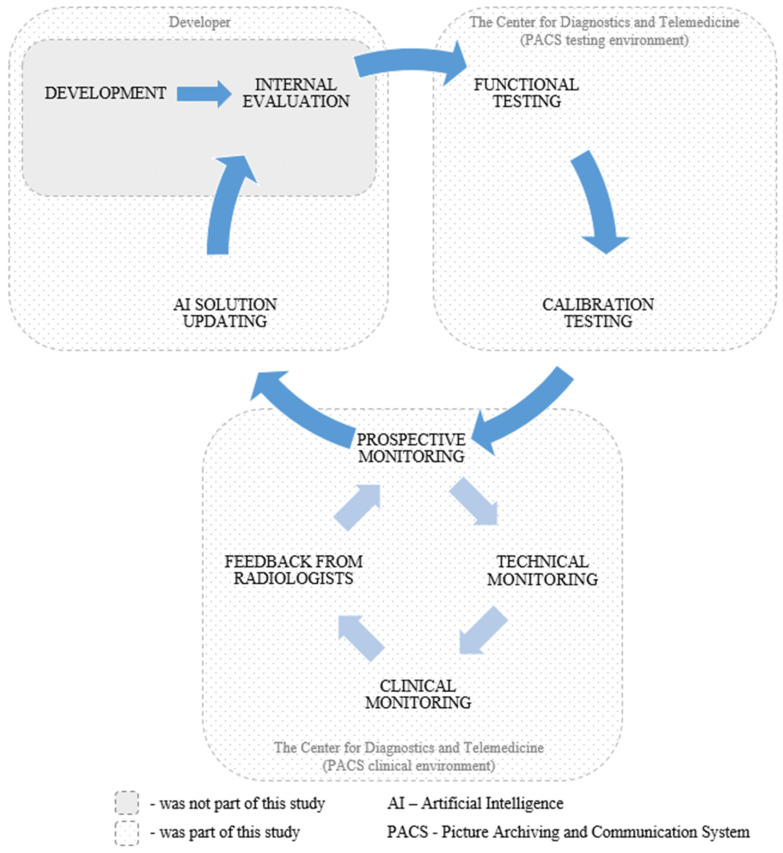
Overview of the study design.

**Figure 3:**
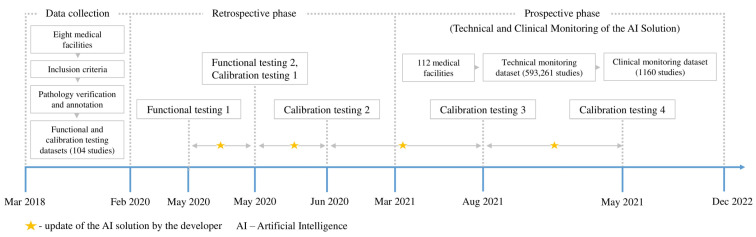
Overview of the study timeline.

This study employed a retrospective and prospective multicenter cohort design. The external evaluation of the AI solution employed our previously published methodology [55]. This study encompassed functional testing, calibration testing, technical monitoring, clinical monitoring, radiologist feedback collection, and AI model updates by the developer. An overview of each testing stage is presented in Table 1. Visual representations of the study design and timeline are presented in Figure 2 and Figure 3, respectively. This study adhered to the guidelines outlined in the Checklist for Artificial Intelligence in Medical Imaging (CLAIM) to ensure transparency and reproducibility [56]. The datasets used for external evaluation and the testing and monitoring methodology are described in detail below.

### 2.4. External Data

This study utilized four datasets: two retrospective (functional and calibration testing datasets) and two prospective (technical and clinical monitoring datasets). Detailed information about the datasets is provided below.

#### 2.4.1. Data Sources and Collection Period

Retrospective data for functional and calibration testing datasets were aggregated from eight medical facilities in Moscow (City clinic №8, City clinic №22, City clinic №36, Diagnostic center №3, Clinical diagnostic center №1, Clinical diagnostic center №4, Clinical diagnostic center №121, M.P. Konchalovsky City clinical hospital №3) between 6 March 2018 and 3 February 2020. Prospective data for technical and clinical monitoring datasets were collected from 112 medical facilities in Moscow between 5 March 2021 and 31 December 2022. The extensive data collection was made possible by the Moscow Radiology Reference Center integrated with the Moscow PACS.

#### 2.4.2. Eligibility Criteria

Inclusion criteria for all datasets were (1) female sex; (2) age ≥ 18 years; and (3) mammograms available in craniocaudal (CC) and mediolateral oblique (MLO) views. Studies with insufficient image quality were excluded using the PGMI (Perfect, Good, Moderate, Inadequate) scale. Studies classified as inadequate included cases such as partial or incomplete mammography, incorrect exposure, blur, and tissue overlapping.

#### 2.4.3. Anonymization Methods and Data Preprocessing

To comply with the applicable regulations [57], all datasets were anonymized according to the Confidentiality Profile Attributes [58]. Four anonymization methods (deletion, cleaning, replacement, and identifier generation) were applied to 433 Digital Imaging and Communications in Medicine (DICOM) tags within the datasets. The anonymization method was documented in the “[0x0012, 0x0063]” tag. In addition, pseudo-anonymization of studies was employed to replace the original study identifier with a randomly generated one. Anonymization was performed using a dataset preparation platform [59]. No other data preprocessing methods were used.

#### 2.4.4. Equipment Manufacturers and Image Acquisition Protocol

The functional and calibration datasets included mammography data acquired from General Electric, TrioDM, and Fujifilm units. The technical and clinical monitoring datasets included equipment from ten manufacturers, including General Electric (*n* = 64), Medical Technologies LTD (*n* = 27), Fujifilm (*n* = 13), etc. Automatic adjustment of imaging parameters, including exposure time, tube voltage, and tube current, was available across all the mammography units. The studies utilized no views other than CC and MLO.

#### 2.4.5. Data Subsets and Sample Size Justification

The functional testing dataset contained four studies derived from the preliminary testing design. The purpose of the functional testing was to evaluate the main functions of the AI model without using quantitative metrics. The limited dataset size also expedited the feedback for the developer. The calibration dataset encompassed 100 studies, with the sampling rationale found in previous publications [60,61]. The calibration dataset is currently available in the public domain [62]. During the technical monitoring, 601,609 studies were sent to the AI model for processing (178,402 studies in 2021 and 423,207 in 2022). For several reasons, the AI model did not process 8348 studies. The final size of the technical monitoring dataset was 593,261 studies. The clinical monitoring dataset contained 1160 studies derived from the technical monitoring dataset each month. During the first ten months of the clinical monitoring (March to December 2021), 20 studies were sampled each month. In the following year (January to December 2022), the monthly sample size increased to 80 studies as the situation with resources for the testing organization had improved. The samples were formed randomly. The sampling rationale may be found in our previous work [63].

#### 2.4.6. Experts, Annotation, and Reference Standard

Two expert radiologists, each with over five years of mammography experience, annotated the studies from the functional and calibration datasets. These radiologists had access to patients’ electronic medical records, including clinical information, prior mammograms, and biopsies conducted within the 12 months following each mammogram. Inclusion required consensus between both specialists; therefore, inter- and intra-reader variabilities were not assessed. The annotation classified studies into two classes (“with pathology” and “without pathology”) according to the Breast Imaging Reporting and Data System (BI-RADS) guidelines [64]. Of these, the “with pathology” class included studies categorized as BI-RADS 3, 4, and 5, while the “without pathology” class consisted of studies interpreted as BI-RADS 1 or 2 [65]. For malignant cases, histological verification and consensus agreement established the ground truth, while consensus agreement alone determined the ground truth for benign cases. Pathology reports were sourced from the Moscow Cancer Registry [66].

During the technical monitoring in the clinical setting, the AI model performed the initial reading, followed by the radiologist’s (i.e., the end user) second reading. The Experiment was designed to replace a second radiologist with an AI system registered as a medical device in compliance with the applicable regulations.

During the clinical monitoring phase, an expert radiologist reviewed the clinical monitoring dataset. The expert had more than 10 years of mammography experience and was trained in working with AI solutions.

The objectives and prospective design of the technical and clinical monitoring phases employed the radiologist’s conclusion as the ground truth.

#### 2.4.7. Demographic and Clinical Characteristics of the Dataset

The functional testing dataset included four females with a median age of 61 years (range: 56–68). The calibration dataset included 100 females with a median age of 63 years (range: 48–71). The functional and calibration datasets were built using a 50/50% class balance (“with pathology”/”without pathology”). Data types of the technical and clinical monitoring datasets are presented in Table 2.

### 2.5. Testing and Monitoring Methodology

#### 2.5.1. Technical Integration of the AI Solution into the PACS

The AI model was integrated into the testing environment of the unified Moscow PACS for external validation. Anonymized studies from static functional and calibration datasets were forwarded to the AI. The previous section provides full details about both datasets. The AI model processed each study and generated an additional series of images and a Digital Imaging and Communications in Medicine Structured Report (DICOM SR). The model also dispatched a Kafka message in JavaScript Object Notation (JSON) format containing the numerical probability of pathological findings for each study. The Kafka messaging framework enabled communication between the AI model and the PACS for seamless data exchange and integration.

#### 2.5.2. Functional Testing

An expert radiologist with over five years of experience in mammography and specialization in radiological AI testing conducted the initial evaluation of the AI model. The functional testing protocol comprised a two-step assessment. First, the radiologist examined the AI’s core functionality as per the Baseline Functional Requirements for radiological AI models (publicly available) [67]. These requirements specify essential criteria governing model operations, including specifications for DICOM SR and additional series, as detailed in Table 3.

Afterwards, the radiologist performed a comprehensive assessment of the AI model from a clinical perspective. This included detailed analysis of the AI’s ability to detect abnormalities such as masses, calcifications, and enlarged lymph nodes. Table 4 shows outcomes of the evaluation performed in line with the Baseline Diagnostic Requirements for mammographic AI (also in the public domain) [67]. Distinguishing between the two types of requirements for AI deliverables is necessary because the Baseline Functional Requirements apply to all clinical AI models, whereas the Baseline Clinical Requirements are task-specific and depend on the model’s intended purpose.

Once the AI model met the Baseline Functional and Diagnostic Requirements, the functional testing was considered passed. The purpose of this testing stage was to filter out various AI models in their early development phase. This approach prevented resource wastage on testing unfinished AI models.

#### 2.5.3. Calibration Testing: Methodology and Statistics

The calibration testing compared finding probability scores generated by the AI model against the annotated calibration dataset as ground truth. This comparison evaluated several diagnostic accuracy metrics, including the area under the curve (AUC), accuracy, sensitivity, specificity, precision, and *F*1 score [68,69]. These metrics utilized the standard classification into true positives (*TP*s), true negatives (*TN*s), false positives (*FP*s), and false negatives (*FN*s):

$$Accuracy=\frac{TP+TN}{TP+FP+TN+FN}$$

$$Sensitivity=\frac{TP}{TP+FN}$$

$$Specificity=\frac{TN}{TN+FP}$$

$$Precision=\frac{TP}{TP+FP}$$

$$F1\;score=\frac{TP}{TP+0.5\times (FP+FN)}$$

To assess the diagnostic performance of the AI model, a receiver operating characteristic (ROC) curve was constructed. This diagram plots the TP rate (sensitivity) against the FP rate (1 − specificity) across various thresholds. The AUC represents the model’s ability to distinguish between positive and negative cases, with values ranging from 0 to 1. The optimal classification threshold was identified using the Youden index method, which maximizes the biomarker’s discriminative ability by balancing sensitivity and specificity [70]. The AI probability score determined class assignment: “no pathology” for scores below the threshold and “with pathology” for scores equal to or above the threshold. The calibration testing was considered successful based on the two criteria presented in Table 1. The AUC threshold of 0.81 was selected based on empirical findings that matched the lower limit of the radiologist’s AUC confidence interval [55]. When the model met or exceeded the thresholds, the calibration testing was considered successful. Otherwise, we recommended that the developer update the AI model and repeat the calibration testing.

#### 2.5.4. Technical Monitoring: Methodology and Statistics

During the next phase, the AI model was integrated into the PACS clinical environment, enabling real-time analysis of anonymized studies acquired in the participating medical facilities. The technical monitoring methodology assessed technical defects originating from the AI model. Technical defects were categorized into two groups: group A (no AI output) and group B (incomplete image analysis). Group B included cases where the AI model analyzed only one, two, or three of the four images comprising a standard mammogram (MLO and CC views for the right and left breasts). Technical defects were monitored on a monthly basis. To assess the defect level in each group, the following ratio was used:

$$Technical\;defect\;level=\frac{D}{S}\times 100\%$$

Here, *D* represents the number of processed studies with technical defects, and *S* represents the total number of studies submitted to the AI model over one month. The average defect level was calculated using the arithmetic mean. To visualize changes over time, we used a linear regression equation to construct a trend line representing the average defect level:

y=ax+b

where *y* represents the average technical defect level, *x* represents the month of observation, and *b* represents the line’s slope. For each group, the technical defect threshold was set to 10% [55]. If the threshold was exceeded, the developer was advised to investigate possible causes and update the AI model.

#### 2.5.5. Clinical Monitoring: Methodology and Statistics

The clinical monitoring methodology involved expert radiologist review. During months 1–10 of monitoring, the expert provided a qualitative assessment of AI outputs with screenshots. In 2022, quantitative assessment was introduced, where the radiologist assigned scores on a scale from 0 to 1 for both graphical mask localization and the DICOM SR interpretation generated by the AI. A score of 0 indicated a false positive, while a score of 1 indicated agreement with the AI results (Table 5).

The mean localization and interpretation scores across all studies in the clinical monitoring sample were calculated using the arithmetic mean. The overall clinical assessment score was derived from these means using the following formula:

$$Clinical\;assessment\;score=\frac{L+I}{2}\times 100\%$$

where *L* represents the mean localization score, and *I* represents the mean interpretation score. Clinical monitoring was conducted by one radiologist at a time. However, due to staff turnover, the radiologist was replaced during the 15th month of monitoring, potentially affecting the results. At the end of each month, both technical and clinical monitoring reports were sent to the developer.

#### 2.5.6. Collecting Feedback from Radiologists and Developers

This study also gathered feedback from the end users (radiologists) of AI in routine clinical practice. Feedback was collected through the dedicated interface in the radiologist workstation, which provided two response options: (1) I agree with the AI outputs; (2) I disagree with the AI outputs. This feedback served as a preliminary assessment of the agreement rate and was forwarded to the developer. The end users participated in the study voluntarily. We also collected written feedback from the developer.

#### 2.5.7. Updating the AI Model—Developer’s Perspective

The developer updated the AI models in two scenarios: (1) testing outcomes were unsatisfactory, and (2) during testing to enhance performance. The first scenario required re-testing to assess update effectiveness. In the second scenario, testing continued without interruption, allowing real-time monitoring of model performance. At the same time, updates were categorized into two types: (1) architectural updates that introduced changes to the model architecture and modified the original functionality, and (2) rectification of coding errors without modifying the architecture or the core functionality. The architectural updates involved comprehensive calibration testing to evaluate the diagnostic accuracy of the updated model. On the contrary, repeated calibration testing post code rectification was considered redundant. Thus, the method for assessing the effectiveness of non-architectural updates was the approach of watchful waiting and continued testing and monitoring. Finally, for both update types, the developer notified testers about version updates and provided change documentation.

## 3. Results

### 3.1. Functional Testing and Initial Update of the AI Model

**Figure 4:**
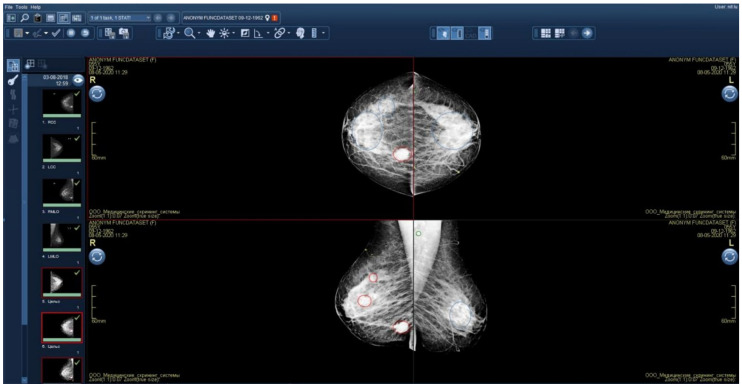
Additional image series with graphical masks highlighting pathological findings.

Initial functional testing of the AI model (version 0.14.0) revealed two inconsistencies with the Baseline Functional Requirements: (1) absence of a “For research purposes only” label in both DICOM SR and additional image series, and (2) using color graphic masks to highlight pathological findings (Figure 4). Color masks (2) were considered inconsistent because they are difficult to distinguish on monochrome mammography monitors. We provided the developer with a functional testing report with recommendations to address these issues. After implementing the requested changes, re-testing we undertaken to make sure that both discrepancies had been resolved.

### 3.2. Calibration Testing and Subsequent Update of the AI Solution

**Figure 5:**
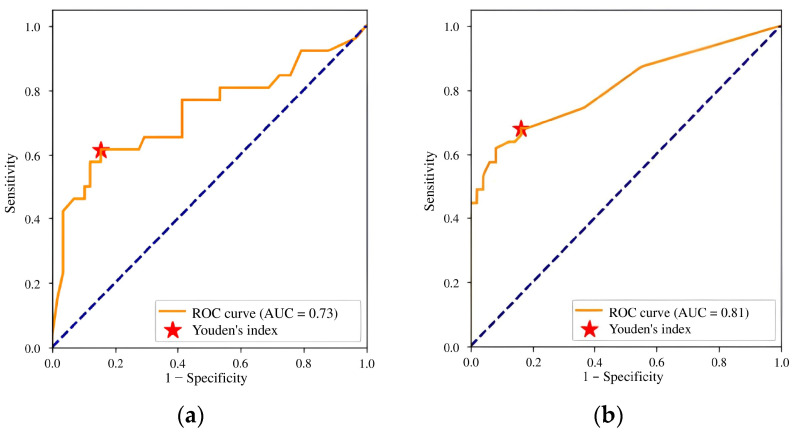
ROC curves for (**a**) version 0.14.0 and (**b**) version 0.15.0 of the Celsus Mammography AI model: results of the first and second calibration tests.

Initial calibration testing of the AI model yielded promising results; however, the AUC and relative accuracy deviation fell below the established thresholds (Figure 5a, Table 6). After identifying these discrepancies, we provided the developer with a calibration testing report and recommendations for improvement. Over the following month, the developer made several modifications, including expanding the training dataset, enhancing image preprocessing, adding a channel for image windowing and histogram equalization, introducing a head module for assessing input image quality, updating the backbone module to ResNeSt [52], switching from standard RoIPooling to precise RoIPooling [71], and modifying the activation function.

The second calibration testing of model version 0.15.0 demonstrated an improvement in diagnostic accuracy. The AUC increased to 0.81, meeting the threshold criteria, and no metric deviated downward by more than 10% compared to the claimed values (Figure 5b, Table 6). These outcomes enable integration of the AI model into the PACS clinical environment for prospective monitoring.

### 3.3. Technical Monitoring Results

**Figure 6:**
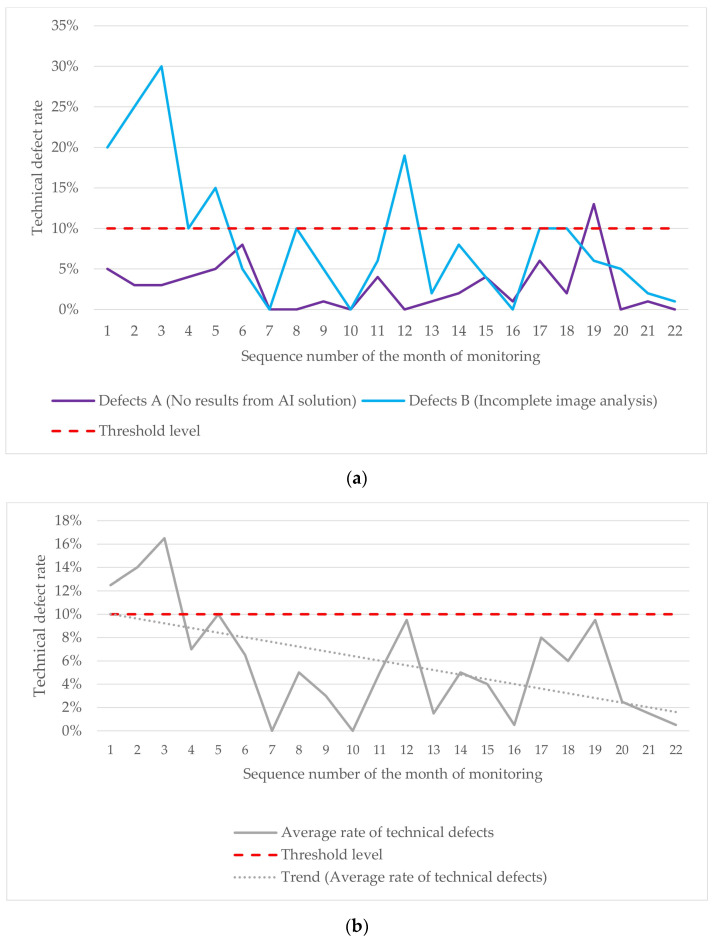
(**a**) Variations in A and B defects during the technical monitoring; (**b**) changes in the average rate of technical defects observed during the technical monitoring.

The technical defect monitoring plot highlights several instances where the technical defects exceeded the established threshold (Figure 6a). The average line demonstrates a downward trend in technical defects, indicating improved model stability (Figure 6b). Technical defects peaked during the initial monitoring phase (months 1–5), with additional spikes in months 12 and 19. Most defects fell into group B, corresponding to incomplete image analysis. Key contributing factors included mammography unit configuration problems, incorrect examination parameters set by technicians, PACS technical issues, and AI developer errors.

### 3.4. Clinical Monitoring Results

**Figure 7:**
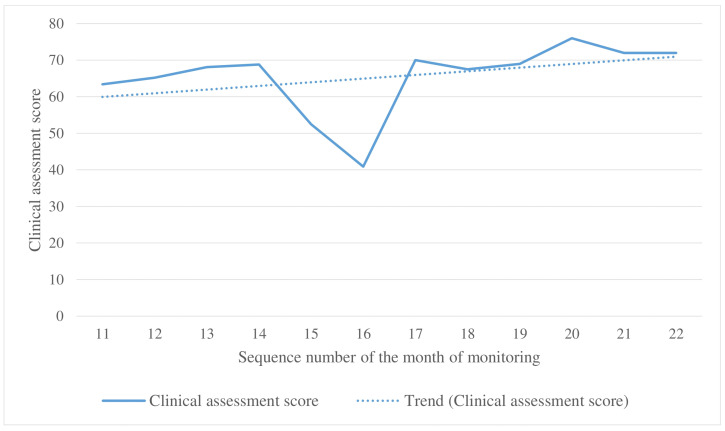
Changes in the clinical assessment score during the clinical monitoring.

The clinical evaluation plot shows a steady upward trend (Figure 7). A decrease in the clinical assessment score during months 15–16 was attributed to the replacement of the evaluating expert radiologist. 

**Figure 8:**
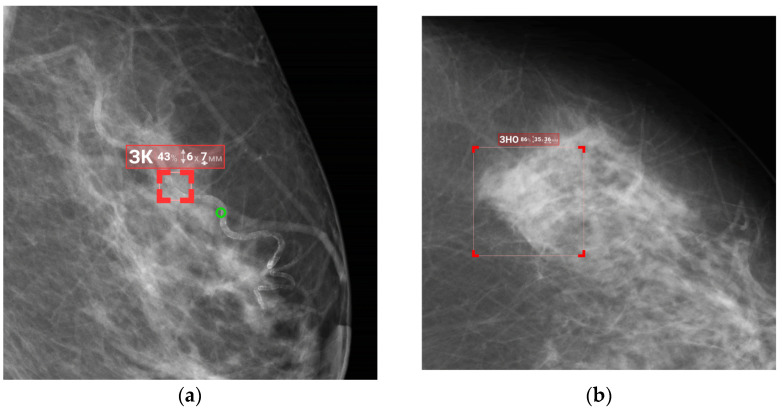
False-positive findings by the AI model: (**a**) benign vascular calcifications misclassified as malignant calcification, and (**b**) normal fibroglandular tissue misclassified as a malignant mass.

**Figure 9:**
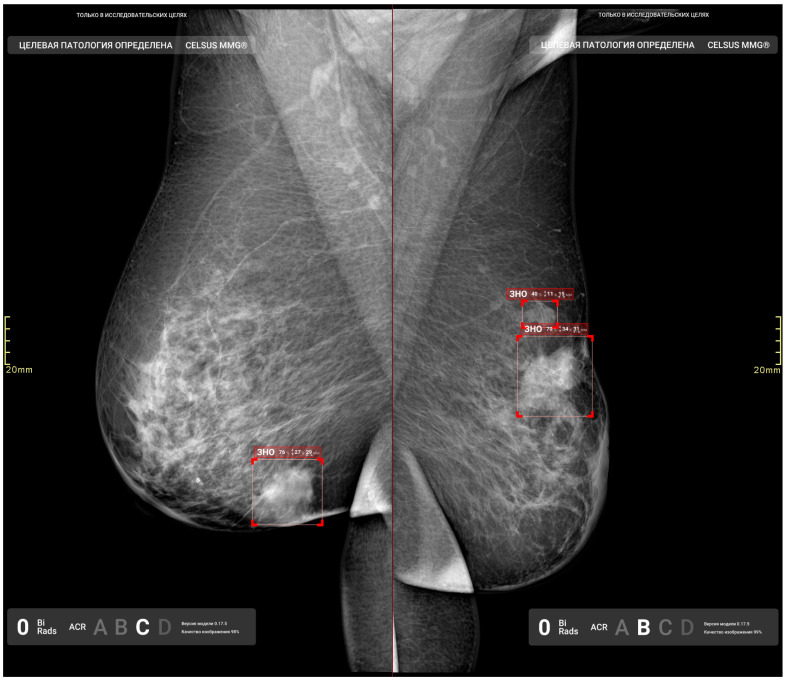
True-positive results from the AI model, illustrating the detected irregular masses with indistinct margins in the right and left breasts.

Figure 8 and Figure 9 illustrate false positives and true positives identified by an expert radiologist. The color-coded boxes represent predicted sites of potential abnormalities. Red boxes marking malignancy sites contain text and numbers indicating the class name (e.g., ‘malignant mass’ or ‘malignant calcification’), class probability, and physical dimensions.

### 3.5. Updates to the AI Model During Prospective Technical and Clinical Monitoring

**Figure 10:**
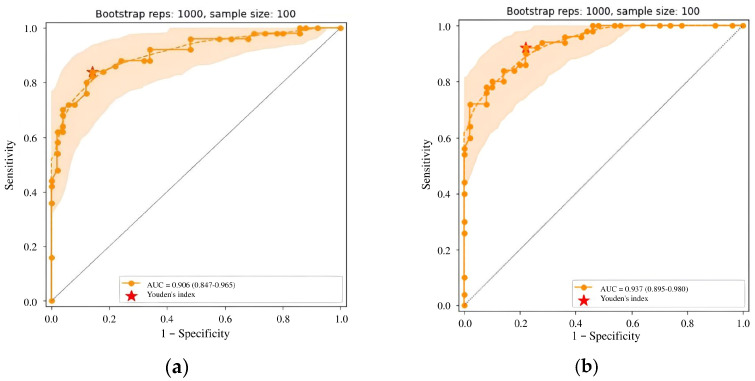
(**a**) ROC curve for Celsus Mammography (version 0.16.0) from the third calibration test; (**b**) ROC curve for Celsus Mammography (version 0.17.0) from the fourth calibration test.

Throughout the prospective phase, the developer implemented the third and fourth updates to the AI model. The third update, installed during month 9 into monitoring, introduced a new class of findings—fibrocystic changes—and a classification system based on the BI-RADS categorization. These updates were driven by radiologist feedback requesting functionality relevant to the clinical setting. Additionally, several architectural adjustments were made. These included a revised approach to meta-model training to enhance the probability assessment and an expanded training dataset. Following these changes, the model version was updated to 0.16.0. A third calibration testing demonstrated a slight improvement in diagnostic accuracy (see Table 7 and Figure 10a).

The fourth update took place in month 15 into the monitoring. It introduced further enhancements, including new classes of findings (inverted nipple and skin thickening), PGMI-based quality classification, and breast quadrants to enhance abnormality localization. The neural network architecture was transitioned to D2Det [72]. AI training was enhanced with new local augmentations and gradient accumulation techniques, while a new meta-model enabled study categorization. The training dataset was also expanded. Diagnostic accuracy metrics for AI model version 0.17.0 demonstrated further improvement (see Table 7 and Figure 10b).

### 3.6. Feedback from Radiologists and the Developer

A total of 8406 feedback responses were collected from 33 end users. The radiologists agreed with the model in 6661 cases (79.2%), and disagreed in 1745 cases (20.8%). Feedback from the AI developer on completed tests is available in Supplementary Materials S2.

### 3.7. Additional Study Results

In January 2023, the Moscow compulsory health insurance system introduced a new reimbursable service, “Description and Interpretation of Mammography Using Artificial Intelligence,” powered by the Celsus AI solution [73]. The integration was preceded by the fifth calibration test. Table 8 presents the results, demonstrating a slight variation in trends compared to the previous calibration testing.

## 4. Discussion

### 4.1. Discussion of the Study Results

This study demonstrates the evolution of a mammographic AI model through testing and monitoring following a lifecycle-based methodology [55]. Our findings show that continuous testing and monitoring significantly enhance the model performance through collaboration among developers, testers, and radiologists. Key improvements include expanded functionality, higher diagnostic accuracy, and improved performance stability.

The initial functional testing identified critical discrepancies that could impede the model’s clinical applicability, specifically non-compliance with the requirements related to labeling and display requirements for AI outputs. Prompt rectification of these issues, followed by successful re-testing, exemplifies the critical importance of functional validation prior to deployment to clinical environments. Unique open-access functional and diagnostic requirements for radiological AI solutions standardize the testing and provide developers with insights for effective integration [67], a matter the developer drew attention to during the course of this study (Supplementary Materials S2). Radiological AI standardization remains a critical topic. Several initiatives were presented by ACR’s Data Science Institute (DSI) Use Cases and the ACR Recognized Center for Healthcare-AI (ARCH-AI) [57]. Most existing standards emphasize ethical and legal aspects rather than technical and clinical requirements.

Further calibration testing revealed inadequate AUC diagnostic accuracy and insufficient sensitivity. Expanding the training dataset and refining the model architecture led to a significant performance improvement. Version 0.15.0 demonstrated a higher AUC of 0.81, achieving 0.94 in version 0.17.0. These adjustments demonstrate the applicability of the lifecycle-based testing and refinement of AI solutions. A recent systematic review on the external validation of mammographic AI models demonstrated an AUC range of 0.71 to 0.96, so our results are consistent with those of other authors [74]. However, the inherent heterogeneity of AI architectures and datasets complicates precise comparisons of diagnostic accuracy across scholarly papers.

Initially, technical monitoring revealed a high defect rate that did not match the stability claims. Further updates led to an unsteady decline in defect rates, demonstrating improved stability. Group B defects (incomplete image analysis) highlighted multiple contributing factors, including human error and technical issues with the PACS and AI. In earlier studies, we demonstrated the prevalence of incorrect DICOM tag entries in chest radiography examinations [75], leading us to suggest that comprehensive technician training could address these discrepancies and improve the adherence with acquisition protocols. A recent systematic review of AI errors in mammography included only one publication focusing on technical rather than clinical errors [76], which indicates that this topic deserves further attention.

Clinical monitoring results revealed an overall improvement in assessment scores, despite personnel-related fluctuations. The presence of false-positive and false-negative results necessitates continuous refinement of AI algorithms alongside experienced radiologists validating AI outputs to improve patient safety and diagnostic accuracy. Although the application of AI in clinical practice is in its early stages, the recent years have witnessed an emergence of prospective studies of mammographic AI [77,78]. Both studies demonstrate increased or stable cancer detection rates compared to the standard approach. However, methodological limitations may present. For example, both radiologists were aware of the AI results when discussing the disputed cases, which may introduce interpretation bias [77]. Since our research did not focus on nuances of AI performance in routine mammography, the prospective phase was limited to binary feedback from end user (agree/disagree) and expert radiologist reviews. Thus, the methodology for prospective studies of radiological AI offers valid contributions to the field and is discussed in this paper.

The end user feedback revealed high radiologist–AI agreement (79.2%), which is promising for the broader introduction of AI into the clinical environment. However, the 20.8% disagreement rate points towards the areas requiring further model training or refinement. Engaging radiologists in the development cycle fosters a collaborative environment and enriches the clinical context, which enhances the AI’s diagnostic capabilities [35,79]. Developer feedback highlighted the testing methodology as a valid contribution to the development and refinement of the AI model. Most of the developer’s responses indicate that third-party testing motivated further improvement (Supplementary Materials S2).

Our findings also showcase the successful integration of the AI model into the compulsory health insurance system, marking a significant milestone in real-world application. Although our previous observation suggested no statistically significant differences in performance between AI and radiologists [73], monitoring AI within clinical workflows is essential for long-term success.

Despite the limited literature on testing methodologies for radiological AI [80,81,82,83], our study aligns with frameworks such as Jorge Cardoso et al.’s Radiology AI Safety, an End-to-end lifecycle approach (RAISE) addressing quality concerns across regulatory, clinical, technical, and ethical domains [46]. While they encompass a broader scope, our methodology provides a narrow perspective on clinical and technical testing.

AI-related publications can be classified into four domains—Plan, Do, Study, Act—with Plan being the most common (rationale for using specific tools, ethical issues, transparency, etc.) [15]. Publications devoted to the Do (appropriate technical expertise, integration into existing workflow) and Act (algorithm improvement for better performance) domains are necessary for deploying AI in clinical practice [84]. We hope this study will contribute to the global adoption of AI in healthcare.

### 4.2. Study Limitations

Several limitations warrant discussion. First, we did not collect various clinical data or register clinical outcomes, such as the cancer detection rate, recall rate, and interval cancers. While this was consistent with our study aims, it may limit the result interpretability. Second, despite a large volume of test data, diagnostic accuracy metrics were calculated using only a calibration dataset of 100 studies. Additionally, the geographical coverage was limited to Moscow, which may also impact result generalizability. Finally, we did not address potential biases associated with AI utilization, which could further affect generalizability.

### 4.3. Future Prospects

Future research directions include expanding testing datasets and investigating the clinical outcomes and biases related to the AI solution’s implementation. Further applications of the testing and monitoring methodology to other AI solutions, along with exploration of prospective clinical monitoring frameworks, are necessary to enhance the robustness of AI implementations in healthcare.

## 5. Conclusions

Our study demonstrates the importance of a structured lifecycle-based methodology for enhancing the mammographic AI performance. Through systematic testing and collaboration among stakeholders, we observed notable improvements in diagnostic accuracy, technical stability, and clinical integration. While our findings offer insights into the effectiveness of mammographic AI, inherent limitations highlight the necessity of further research, particularly concerning study design and data diversity. The successful integration of AI within clinical workflows represents a promising step towards advanced diagnostic radiology. By fostering an active dialogue between developers, clinicians, and regulatory bodies, we can harness the full potential of AI to improve patient outcomes and redefine diagnostic practices in healthcare.

## Figures and Tables

**Table 1:** Objectives and criteria for passing the testing and monitoring stages.

№	Stage	Objective	Pass Criteria
1.	Functional testing	Initial evaluation of the AI model’s core functions by radiologists	No discrepancies with the Baseline Functional and Diagnostic Requirements
2.	Calibration testing	Assessing the diagnostic accuracy metrics using an external histopathologically verified dataset; comparing the results with the metrics claimed by the AI developer	AUC ≥ 0.81; decrease in metric performance does not exceed 10% compared to those claimed by the developer (including AUC, accuracy, sensitivity, specificity)
3.	Technical monitoring	Evaluating the performance stability of the AI model during operation in routine settings	The proportion of studies with technical defects is less than 10% of the monthly study flow
4.	Clinical monitoring	Assessing the clinical effectiveness of the AI model in routine settings	NA *
5.	Collecting feedback from radiologists	Gathering feedback from radiologists who use the AI model in routine practice	NA *

AI—artificial intelligence, AUC—area under the curve. * Note: Since the radiologist’s view is subjective, the results of clinical monitoring and feedback data should be considered as a recommendations rather than instructions. Furthermore, the diagnostic accuracy was primarily evaluated with calibration testing.

**Table 2:** Data types in the technical and clinical monitoring datasets.

Data Type	Technical Monitoring Dataset	Clinical Monitoring Dataset
Years of acquisition	2021–2022	2021–2022
Total patients	593,261	1160
Age group		
≤29	271	0
30–39	8758	14
40–49	139,911	290
50–59	151,488	291
60–69	179,042	321
70–79	91,889	199
≥80	21,902	45
BI-RADS category		
BI-RADS 1	118,672	190
BI-RADS 2	383,053	742
BI-RADS 3	37,268	109
BI-RADS 4	13,273	38
BI-RADS 5	1771	4
BI-RADS 6	1013	5
NA	38,211	72
Medical facilities	112	96
Equipment manufactures	10	10

BI-RADS—Breast Imaging Reporting and Data System, NA—not available.

**Table 3:** Baseline Functional Requirements for outputs of radiological AI models.

№	Evaluation Parameter
Requirements for Additional Image Series	
1	Additional series contains a modified image regardless of the presence of pathological findings
2	Additional series contains graphical masks highlighting a target finding
3	Graphical masks do not extend beyond the target organ
4	For each finding, the graphical mask is labeled by color-coding or a numerical identifier
5	All image series (views) are processed
6	Images in the additional series are not cropped
Requirements for DICOM SR	
1	DICOM SR template (including modality, region of interest, unique study identifier, clinical task for the AI model, technical parameters, and a brief user manual)
2	The DICOM SR template meets the Baseline Diagnostic Requirements
Requirements for additional image series and DICOM SR	
1	Additional series and DICOM SR generated by the AI model for each processed study
2	Additional series and DICOM SR contain the name and version of the AI model
3	Additional series and DICOM SR contain the date and time of analysis completion
4	Additional series and DICOM SR contain the “For research purpose only” notification
7	No contradicting data in the additional series and DICOM SR (for example, report contains no impression of the labeled findings)
Other	
1	No other critical errors in AI operation (including error messages, non-diagnostic radiology report, poor graphical mask visibility, overly long loading time of additional series, etc.)

DICOM SR—Digital Imaging and Communications in Medicine Structured Report (DICOM SR), AI—artificial intelligence.

**Table 4:** Baseline Diagnostic Requirements for mammographic AI outputs detecting signs of breast cancer.

№	AI Response	Response Format	Response Form
1	Detection, segmentation, and classification (benign/malignant) of masses	Graphical mask, text	DICOM, DICOM SR, Apache Kafka message
2	Detection, segmentation, and classification (benign/malignant) of calcifications		
3	Detection and segmentation of enlarged lymph nodes		
4	ACR category of breast density for each breast	Text	DICOM SR
5	Probability of breast cancer in the entire study	Number	Apache Kafka message

AI—artificial intelligence, DICOM (SR)—Digital Imaging and Communications in Medicine (Structured Report), ACR—American College of Radiology.

**Table 5:** Clinical evaluation criteria for mammographic AI models.

Evaluation Criteria	Description	Score	
		Localization	Interpretation
Agreement	AI accurately labeled and interpreted all abnormalities	1	1
Incorrect assessment	Not all target abnormalities detected	0.5	0.5
False positive	Detected abnormalities that were not there	0.25	0.25
False negative	Failure to detect target abnormalities	0	0

**Table 6:** Diagnostic accuracy metrics of the Celsus Mammography AI model: results of the first and second calibration tests.

Metric	CalT1 (Claimed)	CalT1 (Obtained)	Rel. Diff. (%)	CalT 2 (Claimed)	CalT 2 (Obtained)	Rel. Diff. (%)
AUC	NA	**0.73**	NA	NA	0.81	NA
Acc.	0.71	0.73	+3%	0.71	0.76	+7%
Sens.	0.72	0.62	**−14%**	0.72	0.68	−6%
Spec.	0.68	0.84	+24%	0.68	0.84	+24%
Precision	NA	0.79	NA	NA	0.81	NA
F1 score	NA	0.70	NA	NA	0.74	NA

CalT—calibration testing, AUC—area under the curve, Acc.—accuracy, Sens.—sensitivity, Spec.—specificity, Rel. Diff.—relative difference. Metrics that do not meet the threshold criteria are highlighted in bold.

**Table 7:** Diagnostic accuracy metrics of the Celsus Mammography seen in the third and fourth calibration tests.

Metric	CalT3 (Claimed)	CalT3 (Obtained)	Rel. Diff. (%)	CalT4 (Claimed)	CalT4 (Obtained)	Rel. Diff. (%)
AUC	0.857	0.91	+6%	0.886	0.94	+6%
Acc.	0.57	0.85	+27%	0.82	0.85	+4%
Sens.	0.87	0.84	−3%	0.82	0.92	+12%
Spec.	0.66	0.86	+30%	0.82	0.78	−5%
Precision	NA	0.86	NA	NA	0.81	NA
F1 score	NA	0.86	NA	NA	0.86	NA

CalT—calibration testing, AUC—area under the curve, Acc.—accuracy, Sens.—sensitivity, Spec.—specificity, Rel. Diff.—relative difference.

**Table 8:** Diagnostic accuracy metrics of the Celsus Mammography version 0.18.0: results of the fifth calibration test.

Metric	CalT5 (Claimed)	CalT5 (Obtained)	Rel. Diff. (%)
AUC	0.89	0.91	+2.3%
Acc.	0.82	0.89	+8.5%
Sens.	0.82	0.85	+3.7%
Spec.	0.82	0.93	+13.4%
Precision	NA	0.91	NA
F1 score	NA	0.88	NA

CalT—calibration testing, AUC—area under the curve, Acc.—accuracy, Sens.—sensitivity, Spec.—specificity, Rel. Diff.—relative difference.

## Data Availability

The public datasets used in this study can be found at https://mosmed.ai/en/datasets/.

## Supplementary Materials

The following supporting information can be downloaded at https://www.mdpi.com/article/10.3390/diagnostics15070822/s1, Supplementary Materials S1. AI model profile; Supplementary Materials S2. Feedback from the AI solution developer.

## References

[1] Katsika L., Boureka E., Kalogiannidis I., Tsakiridis I., Tirodimos I., Lallas K., Tsimtsiou Z., Dagklis T. (2024). Screening for Breast Cancer: A Comparative Review of Guidelines. Life.

[2] Akwo J., Hadadi I., Ekpo E. (2024). Diagnostic Efficacy of Five Different Imaging Modalities in the Assessment of Women Recalled at Breast Screening—A Systematic Review and Meta-Analysis. Cancers.

[3] Ding L., Greuter M.J.W., Truyen I., Goossens M., Van der Vegt B., De Schutter H., Van Hal G., de Bock G.H. (2022). Effectiveness of Organized Mammography Screening for Different Breast Cancer Molecular Subtypes. Cancers.

[4] Nicosia L., Gnocchi G., Gorini I., Venturini M., Fontana F., Pesapane F., Abiuso I., Bozzini A.C., Pizzamiglio M., Latronico A. (2023). History of Mammography: Analysis of Breast Imaging Diagnostic Achievements over the Last Century. Healthcare.

[5] Magnus M.C., Ping M., Shen M.M., Bourgeois J., Magnus J.H. (2011). Effectiveness of Mammography Screening in Reducing Breast Cancer Mortality in Women Aged 39–49 Years: A Meta-Analysis. J. Women’s Health.

[6] Marmot M.G., Altman D.G., Cameron D.A., Dewar J.A., Thompson S.G., Wilcox M. (2013). The Benefits and Harms of Breast Cancer Screening: An Independent Review. Br. J. Cancer.

[7] Nelson H.D., Fu R., Cantor A., Pappas M., Daeges M., Humphrey L. (2016). Effectiveness of Breast Cancer Screening: Systematic Review and Meta-Analysis to Update the 2009 U.S. Preventive Services Task Force Recommendation. Ann. Intern. Med..

[8] Dibden A., Offman J., Duffy S.W., Gabe R. (2020). Worldwide Review and Meta-Analysis of Cohort Studies Measuring the Effect of Mammography Screening Programmes on Incidence-Based Breast Cancer Mortality. Cancers.

[9] Hirani R., Noruzi K., Khuram H., Hussaini A.S., Aifuwa E.I., Ely K.E., Lewis J.M., Gabr A.E., Smiley A., Tiwari R.K. (2024). Artificial Intelligence and Healthcare: A Journey through History, Present Innovations, and Future Possibilities. Life.

[10] Najjar R. (2023). Redefining Radiology: A Review of Artificial Intelligence Integration in Medical Imaging. Diagnostics.

[11] Avanzo M., Stancanello J., Pirrone G., Drigo A., Retico A. (2024). The Evolution of Artificial Intelligence in Medical Imaging: From Computer Science to Machine and Deep. Learning. Cancers.

[12] Pinto-Coelho L. (2023). How Artificial Intelligence Is Shaping Medical Imaging Technology: A Survey of Innovations and Applications. Bioengineering.

[13] Obuchowicz R., Strzelecki M., Piórkowski A. (2024). Clinical Applications of Artificial Intelligence in Medical Imaging and Image Processing—A Review. Cancers.

[14] Karalis V.D. (2024). The Integration of Artificial Intelligence into Clinical Practice. Appl. Biosci..

[15] Khan S.D., Hoodbhoy Z., Raja M.H.R., Kim J.Y., Hogg H.D.J., Manji A.A.A., Gulamali F., Hasan A., Shaikh A., Tajuddin S. (2024). Frameworks for Procurement, Integration, Monitoring, and Evaluation of Artificial Intelligence Tools in Clinical Settings: A Systematic Review. PLoS Digit. Health.

[16] Maleki Varnosfaderani S., Forouzanfar M. (2024). The Role of AI in Hospitals and Clinics: Transforming Healthcare in the 21st Century. Bioengineering.

[17] Al-Karawi D., Al-Zaidi S., Helael K.A., Obeidat N., Mouhsen A.M., Ajam T., Alshalabi B.A., Salman M., Ahmed M.H. (2024). A Review of Artificial Intelligence in Breast Imaging. Tomography.

[18] Zhu Z., Sun Y., Honarvar Shakibaei Asli B. (2024). Early Breast Cancer Detection Using Artificial Intelligence Techniques Based on Advanced Image Processing Tools. Electronics.

[19] Shamir S.B., Sasson A.L., Margolies L.R., Mendelson D.S. (2024). New Frontiers in Breast Cancer Imaging: The Rise of AI. Bioengineering.

[20] Khalid A., Mehmood A., Alabrah A., Alkhamees B.F., Amin F., AlSalman H., Choi G.S. (2023). Breast Cancer Detection and Prevention Using Machine Learning. Diagnostics.

[21] Carriero A., Groenhoff L., Vologina E., Basile P., Albera M. (2024). Deep. Learning in Breast Cancer Imaging: State of the Art. and Recent. Advancements in Early 2024. Diagnostics.

[22] Adachi M., Fujioka T., Ishiba T., Nara M., Maruya S., Hayashi K., Kumaki Y., Yamaga E., Katsuta L., Hao D. (2024). AI Use in Mammography for Diagnosing Metachronous Contralateral Breast Cancer. J. Imaging.

[23] Wing P., Langelier M.H. (2009). Workforce Shortages in Breast Imaging: Impact on Mammography Utilization. Am. J. Roentgenol..

[24] Kalidindi S., Gandhi S. (2023). Workforce Crisis in Radiology in the UK and the Strategies to Deal. With It: Is. Artificial Intelligence the Saviour?. Cureus.

[25] Kwee T.C., Kwee R.M. (2021). Workload of Diagnostic Radiologists in the Foreseeable Future Based on Recent Scientific Advances: Growth Expectations and Role of Artificial Intelligence. Insights Imaging.

[26] Gastounioti A., Eriksson M., Cohen E.A., Mankowski W., Pantalone L., Ehsan S., McCarthy A.M., Kontos D., Hall P., Conant E.F. (2022). External Validation of a Mammography-Derived AI-Based Risk Model in a U.S. Breast Cancer Screening Cohort of White and Black Women. Cancers.

[27] Yu A.C., Mohajer B., Eng J. (2022). External Validation of Deep Learning Algorithms for Radiologic Diagnosis: A Systematic Review. Radiol. Artif. Intell..

[28] Martin-Noguerol T., Luna A. (2021). External Validation of AI Algorithms in Breast Radiology: The Last Healthcare Security Checkpoint?. Quant. Imaging Med. Surg..

[29] Vasilev Y., Vladzymyrskyy A., Omelyanskaya O., Blokhin I., Kirpichev Y., Arzamasov K. (2023). AI-Based CXR First Reading: Current Limitations to Ensure Practical Value. Diagnostics.

[30] Kim D.W., Jang H.Y., Kim K.W., Shin Y., Park S.H. (2019). Design Characteristics of Studies Reporting the Performance of Artificial Intelligence Algorithms for Diagnostic Analysis of Medical Images: Results from Recently Published Papers. Korean J. Radiol..

[31] Petersson L., Larsson I., Nygren J.M., Nilsen P., Neher M., Reed J.E., Tyskbo D., Svedberg P. (2022). Challenges to Implementing Artificial Intelligence in Healthcare: A Qualitative Interview Study with Healthcare Leaders in Sweden. BMC Health Serv. Res..

[32] Ramwala O.A., Lowry K.P., Cross N.M., Hsu W., Austin C.C., Mooney S.D., Lee C.I. (2024). Establishing a Validation Infrastructure for Imaging-Based Artificial Intelligence Algorithms Before Clinical Implementation. J. Am. Coll. Radiol..

[33] Freeman K., Geppert J., Stinton C., Todkill D., Johnson S., Clarke A., Taylor-Phillips S. (2021). Use of Artificial Intelligence for Image Analysis in Breast Cancer Screening Programmes: Systematic Review of Test Accuracy. BMJ.

[34] Hickman S.E., Baxter G.C., Gilbert F.J. (2021). Adoption of Artificial Intelligence in Breast Imaging: Evaluation, Ethical Constraints and Limitations. Br. J. Cancer.

[35] Dikici E., Bigelow M., Prevedello L.M., White R.D., Erdal B.S. (2020). Integrating AI into Radiology Workflow: Levels of Research, Production, and Feedback Maturity. J. Med. Imaging.

[36] Brady A.P., Allen B., Chong J., Kotter E., Kottler N., Mongan J., Oakden-Rayner L., dos Santos D.P., Tang A., Wald C. (2024). Developing, Purchasing, Implementing and Monitoring AI Tools in Radiology: Practical Considerations. A Multi-Society Statement from the ACR, CAR, ESR, RANZCR & RSNA. Insights Imaging.

[37] Pianykh O.S., Langs G., Dewey M., Enzmann D.R., Herold C.J., Schoenberg S.O., Brink J.A. (2020). Continuous Learning AI in Radiology: Implementation Principles and Early Applications. Radiology.

[38] Sinha S., Lee Y.M. (2024). Challenges with Developing and Deploying AI Models and Applications in Industrial Systems. Discov. Artif. Intell..

[39] Smith A., Severn M. (2022). An Overview of Continuous Learning Artificial Intelligence-Enabled Medical Devices. Can. J. Health Technol..

[40] Wang L., Zhang X., Su H., Zhu J. (2024). A Comprehensive Survey of Continual Learning: Theory, Method and Application. IEEE Trans. Pattern Anal. Mach. Intell..

[41] Harvey H., Heindl A., Khara G., Korkinof D., O’Neill M., Yearsley J., Karpati E., Rijken T., Kecskemethy P., Forrai G. (2019). Deep Learning in Breast Cancer Screening. Artificial Intelligence in Medical Imaging: Opportunities, Applications and Risks.

[42] Gichoya J.W., Thomas K., Celi L.A., Safdar N., Banerjee I., Banja J.D., Seyyed-Kalantari L., Trivedi H., Purkayastha S. (2023). AI Pitfalls and What Not to Do: Mitigating Bias in AI. Br. J. Radiol..

[43] Tejani A.S., Ng Y.S., Xi Y., Rayan J.C. (2024). Understanding and Mitigating Bias in Imaging Artificial Intelligence. Radiographics.

[44] Vrudhula A., Kwan A.C., Ouyang D., Cheng S. (2024). Machine Learning and Bias in Medical Imaging: Opportunities and Challenges. Circ. Cardiovasc. Imaging.

[45] Park S.H., Han K., Jang H.Y., Park J.E., Lee J.G., Kim D.W., Choi J. (2023). Methods for Clinical Evaluation of Artificial Intelligence Algorithms for Medical Diagnosis. Radiology.

[46] Cardoso M.J., Moosbauer J., Cook T.S., Erdal B.S., Genereaux B., Gupta V., Landman B.A., Lee T., Somasundaram E., Summers R.M. RAISE-Radiology AI Safety, an End-to-End Lifecycle Approach.

[47] Ng M.Y., Kapur S., Blizinsky K.D., Hernandez-Boussard T. (2022). The AI Life Cycle: A Holistic Approach to Creating Ethical AI for Health Decisions. Nat. Med..

[48] De Silva D., Alahakoon D. (2022). An Artificial Intelligence Life Cycle: From Conception to Production. Patterns.

[49] Experiment on the Introduction of Artificial Intelligence Technologies. https://telemedai.ru/en/proekty/eksperiment-po-vnedreniyu-tehnologij-iskusstvennogo-intellekta.

[50] Celsus Mammography. https://celsus.ai/en/products-mammography/.

[51] Ren S., He K., Girshick R., Sun J. (2017). Faster R-CNN: Towards Real-Time Object Detection with Region Proposal Networks. IEEE Trans. Pattern Anal. Mach. Intell..

[52] Zhang H., Wu C., Zhang Z., Zhu Y., Lin H., Zhang Z., Sun Y., He T., Mueller J., Manmatha R. ResNeSt: Split-Attention Networks. Proceedings of the IEEE/CVF Conference on Computer Vision and Pattern Recognition.

[53] CELS® Software Registration Certificate for a Medical Device 2021/14449. https://nevacert.ru/reestry/med-reestr/rzn-202114449-46522.html.

[54] The Celsus Medical Decision Support System Has Received a CE Mark. https://celsus.ai/en/news/the-celsus-medical-decision-support-system-has-received-a-ce-mark/.

[55] Vasilev Y.A., Vladzymyrskyy A.V., Omelyanskaya O.V., Arzamasov K.M., Chetverikov S.F., Rumyantsev D.A., Zelenova M.A. (2023). Methodology for Testing and Monitoring Artificial Intelligence-Based Software for Medical Diagnostics. Digit. Diagn..

[56] Mongan J., Moy L., Kahn C.E. (2020). Checklist for Artificial Intelligence in Medical Imaging (CLAIM): A Guide for Authors and Reviewers. Radiol. Artif. Intell..

[57] Avrin D. (2008). HIPAA Privacy and DICOM Anonymization for Research. Acad. Radiol..

[58] DICOM Standards Committee Application Level Confidentiality Profile Attributes. https://dicom.nema.org/medical/dicom/current/output/html/part15.html#table_E.1-1.

[59] Vasiliev Y.A., Vladzimirskyy A.V., Omelyanskaya O.V., Arzamasov K.M., Savkina E.F., Kasimov S.D., Kosov P.N., Ponomarenko A.P., Medvedev K.E., Burtsev T.A. Certificate of State Registration of Computer Program No. 2025610804 Russian Federation. Dataset Preparation Platform: No. 2024691653: Declared 20 December 2024: Published 14 January 2025. https://www.elibrary.ru/item.asp?id=80277623.

[60] Bobrovskaya T.M., Nikitin N.Y., Vladzymyrskyy A.V., Omelyanskaya O.V. (2024). Sample Size for Assessing a Diagnostic Accuracy of AI-Based Software in Radiology. Sib. J. Clin. Exp. Med..

[61] Arzamasov K., Vasilev Y., Zelenova M., Pestrenin L., Busygina Y., Bobrovskaya T., Chetverikov S., Shikhmuradov D., Pankratov A., Kirpichev Y. (2024). Independent Evaluation of the Accuracy of 5 Artificial Intelligence Software for Detecting Lung Nodules on Chest X-Rays. Quant. Imaging Med. Surg..

[62] MosMedData: MMG with and without Signs of Breast Malignancies, Enriched with Clinical Information. https://mosmed.ai/en/datasets/mosmeddata-mmg-s-nalichiem-i-otsutstviem-priznakov-zlokachestvennih-novoobrazovanii-molochnoi-zhelezi-obogaschennii-klinicheskoi-informatsiei/.

[63] Chetverikov S.F., Arzamasov K.M., Andreichenko A.E., Novik V.P., Bobrovskaya T.M., Vladzimirskyy A.V. (2023). Approaches to Sampling for Quality Control of Artificial Intelligence in Biomedical Research. Sovrem. Tehnol. Med..

[64] Spak D.A., Plaxco J.S., Santiago L., Dryden M.J., Dogan B.E. (2017). BI-RADS® Fifth Edition: A Summary of Changes. Diagn. Interv. Imaging.

[65] Chang Sen L.Q., Mayo R.C., Lesslie M.D., Yang W.T., Leung J.W.T. (2018). Impact of Second-Opinion Interpretation of Breast Imaging Studies in Patients Not Currently Diagnosed with Breast Cancer. J. Am. Coll. Radiol..

[66] Kaprin A.D., Chissov V.I., Starinsky V.V., Gretsova O.P., Petrova G.V., Prostov Y.I. (2015). The Information Analytical System for Registration of Cancer Patients in the Russian Federation. P.A. Herzen J. Oncol..

[67] Baseline Functional and Diagnostic Requirements for AI Services. https://mosmed.ai/en/ai/docs/.

[68] Baratloo A., Hosseini M., Negida A., El Ashal G. (2015). Part 1: Simple Definition and Calculation of Accuracy, Sensitivity and Specificity. Emergency.

[69] Hicks S.A., Strümke I., Thambawita V., Hammou M., Riegler M.A., Halvorsen P., Parasa S. (2022). On Evaluation Metrics for Medical Applications of Artificial Intelligence. Sci. Rep..

[70] Ruopp M.D., Perkins N.J., Whitcomb B.W., Schisterman E.F. (2008). Youden Index and Optimal Cut-Point Estimated from Observations Affected by a Lower Limit of Detection. Biom. J..

[71] Jiang B., Luo R., Mao J., Xiao T., Jiang Y. Acquisition of Localization Confidence for Accurate Object Detection. Proceedings of the European Conference on Computer Vision (ECCV).

[72] Cao J., Cholakkal H., Anwer R.M., Khan F.S., Pang Y., Shao L. D2DET: Towards High Quality Object Detection and Instance Segmentation. Proceedings of the 2020 IEEE/CVF Conference on Computer Vision and Pattern Recognition (CVPR).

[73] Vasiliev Y.A., Vladzimirskyy A.V., Arzamasov K.M., Shulkin I.M., Aksenova L.E., Pestrenin L.D., Semenov S.S., Bondarchuk D.V., Smirnov I.V. (2023). The First 10,000 Mammography Exams Performed as Part of the “Description and Interpretation of Mammography Data Using Artificial Intelligence” Service. Manag. Zdr..

[74] Branco P.E.S.C., Franco A.H.S., de Oliveira A.P., Carneiro I.M.C., de Carvalho L.M.C., de Souza J.I.N., Leandro D.R., Cândido E.B. (2024). Artificial Intelligence in Mammography: A Systematic Review of the External Validation. Rev. Bras. Ginecol. Obstet..

[75] Borisov A.A., Arzamasov K.M., Semenov S.S., Vladzimirsky A.V., Vasiliev Y.A. (2024). Investigation of the Capabilities of Algorithms for Automated Quality Assurance of DICOM Metadata of Chest X-ray Examinations. Med. Vis..

[76] Zeng A., Houssami N., Noguchi N., Nickel B., Marinovich M.L. (2024). Frequency and Characteristics of Errors by Artificial Intelligence (AI) in Reading Screening Mammography: A Systematic Review. Breast Cancer Res. Treat..

[77] Dembrower K., Crippa A., Colón E., Eklund M., Strand F. (2023). Artificial Intelligence for Breast Cancer Detection in Screening Mammography in Sweden: A Prospective, Population-Based, Paired-Reader, Non-Inferiority Study. Lancet Digit. Health.

[78] Lång K., Josefsson V., Larsson A.M., Larsson S., Högberg C., Sartor H., Hofvind S., Andersson I., Rosso A. (2023). Artificial Intelligence-Supported Screen Reading versus Standard Double Reading in the Mammography Screening with Artificial Intelligence Trial (MASAI): A Clinical Safety Analysis of a Randomised, Controlled, Non-Inferiority, Single-Blinded, Screening Acc. Lancet Oncol..

[79] Allen B., Gish R., Dreyer K. (2019). The Role of an Artificial Intelligence Ecosystem in Radiology. Artificial Intelligence in Medical Imaging: Opportunities, Applications and Risks.

[80] Waller J., O’connor A., Rafaat E., Amireh A., Dempsey J., Martin C., Umair M. (2022). Applications and Challenges of Artificial Intelligence in Diagnostic and Interventional Radiology. Polish J. Radiol..

[81] Chang J.Y., Makary M.S. (2024). Evolving and Novel Applications of Artificial Intelligence in Thoracic Imaging. Diagnostics.

[82] Brady A.P., Neri E. (2020). Artificial Intelligence in Radiology-Ethical Considerations. Diagnostics.

[83] Allen B., Dreyer K., Stibolt R., Agarwal S., Coombs L., Treml C., Elkholy M., Brink L., Wald C. (2021). Evaluation and Real-World Performance Monitoring of Artificial Intelligence Models in Clinical Practice: Try It, Buy It, Check It. J. Am. Coll. Radiol..

[84] Isa I.G.T., Ammarullah M.I., Efendi A., Nugroho Y.S., Nasrullah H., Sari M.P. (2024). Constructing an Elderly Health Monitoring System Using Fuzzy Rules and Internet of Things. AIP Adv..

