# Supplementary material for: Evolution of an Artificial Intelligence-Powered Application for Mammography
Source: Diagnostics (Basel). 2025 Mar 24;15(7):822. doi: 10.3390/diagnostics15070822 (PMC11988740; doi:10.3390/diagnostics15070822)
Supplement: Supplementary file 1 [file diagnostics-15-00822-s001.zip › diagnostics-3535858-supplementary.pdf]

Supplementary Material 1. AI model profile.

We requested the developer to provide an AI model profile using the CLAIM checklist. The checklist items include Data, Reference Standard, Data Partitions, Model, Training, and Evaluation.

Data

Data sources: The data were acquired from approximately several dozen medical facilities (the exact number of data sources was not recorded). The primary data sources included Kaluga Regional Clinical Cancer Center, Nizhny Novgorod Regional Clinical Cancer Center, CBIS-DDSM dataset.

Inclusion criteria: (1) digital mammograms in the cranial caudal (CC) and mediolateral oblique (MLO) views, (2) female patients over 18 years of age. Exclusion criteria: mammograms with inadequate quality for diagnosis (class I on the PGMI scale).

Data pre-processing: DICOM files were preprocessed using the Pydicom library version 2.3.1. Information extracted from DICOM tags: view (CC or MLO), side (left or right), value of Interest (VOI), LUT parameters. Grayscale inversion of positive radiographic images ensured unified photometric interpretation. A transformation function was applied to each image. If the transformation function DICOM tags were blank, the image was normalized by converting the pixel values to the range from 0 to 1. The image resized to 1000 x 2000 pixels using the Letterbox operation before neural network processing. Annotations were scaled to match the preprocessed image dimensions.

Selection of data subsets: Data subdivision was performed in two steps. First, the mammograms were categorized into three classes: "normal", "benign", and "malignant". Second, the data were classified according to the BI-RADS categories.

Data anonymization: DICOM tags containing personal information (patient name, patient ID, referring physician name, attending physician name, study date, etc.) were removed to comply with regulatory requirements.

Missing data: No missing data were present.

Image acquisition protocol: Details regarding the scanning protocol were not available. To our knowledge, mammography units were capable of automatically selecting imaging protocols appropriate for breast density.

Reference Standard

Each study was annotated by two or three radiologists and served as the reference standard (ground truth). The first radiologist annotated the mammograms, which were subsequently reviewed by a second radiologist. A third specialist was engaged when the first two radiologists could not reach a consensus. Mammograms were included in the training dataset only after the annotators reached a consensus. Images were annotated using a proprietary annotation tool. The annotators received standard quality control instructions. Mammography findings were classified in accordance with the BI-RADS atlas. In total, more than 10 practicing radiologists with 10 to 25 years of experience were engaged. Inter- or intra-rater variability was not assessed, as the images were included in the dataset only upon annotator consensus.

Data Partitions

The data were subdivided into training, validation and test datasets at a ratio of 80/10/10, a commonly used approach for data partitioning. No systematic differences were observed between the data in each partition. Data partitioning was performed at the patient level. The training dataset comprised 3,641 mammograms (818 mammograms from the Kaluga Regional Clinical Cancer Center, 1,573 mammograms from the Nizhny Novgorod Regional Clinical Cancer Center, 1,250 mammograms from the CBIS-DDSM dataset).

Model

The neural network utilized the Faster-RCNN architecture with a ResNet-50 backbone. The following modifications were implemented to enhance the model performance:

1. Batch Normalization was replaced with Group Normalization because the large size of the original mammograms restricted the batch size during training.

2. The number of input image channels was changed from 3 (RGB) to 1 (grayscale).

3. A feature extraction mechanism (“attention”) was incorporated for CC and MLO projections.

4. Additional Region Proposal Network (RPN) blocks and detection heads were added to enable training on the datasets that contained different class sets and annotation rules.

The model identified five classes of abnormalities: malignant lesion, benign lesion, malignant calcifications, benign calcifications, and lymph nodes. Detected abnormalities underwent non-maximum suppression procedures (class-agnostic); cut-off thresholds were set individually for each class. Additionally, convolutional features from the images were used to predict breast density. The probability of an abnormality, such as lesions or calcifications, was derived from object prediction scores. These properties, along with the predicted breast density, were put into the trained meta-model powered by the LightGBM algorithm which calculated the final probability of abnormality. The pipeline for determining the final abnormality probability is shown in Figure S1.

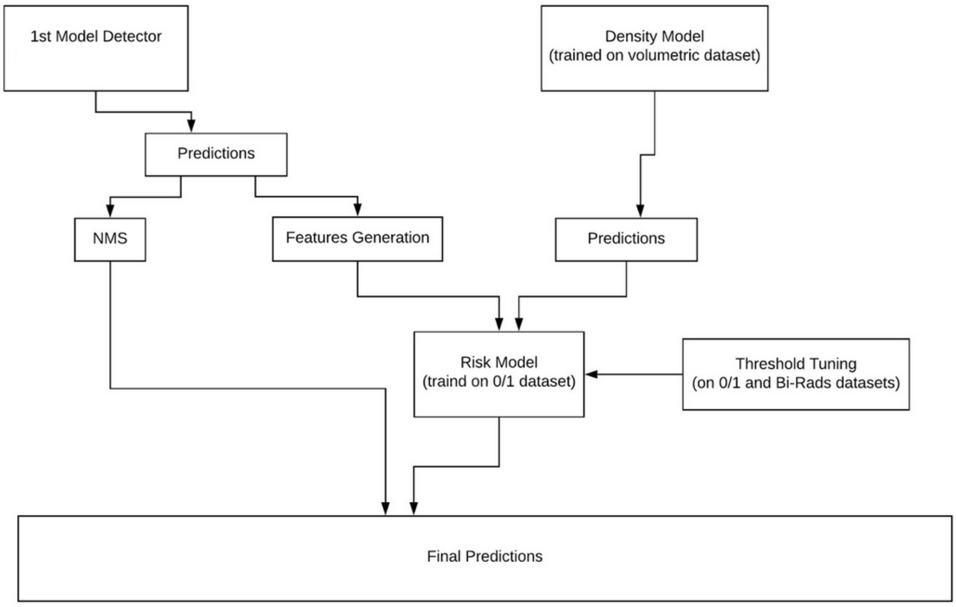

Figure S1. Pipeline for determining the final abnormality probability.

During model deployment, the torch and torchvision software libraries were used. The albumentations library was used to generate augmentations. Model parameters were initialized randomly using a random number initialization method.

Training

The model was trained for 30 epochs, with the 29th epoch selected as the checkpoint due to achieving the highest metrics. The optimization algorithm RAdam (Rectified Adam) was used as the optimizer. No ensembling techniques were used.

Evaluation

We used the following metrics to track the model performance: AUC, Accuracy, Sensitivity, and Specificity. To assess the performance consistency across the training and test datasets, class balance was maintained. This was achieved by ensuring the BI-RADS category ratios matched in both datasets. No statistical measures for significance and uncertainty, robustness or sensitivity analysis, or other methods to enhance explainability or interpretability were employed.

Supplementary Material 2. Feedback from the AI Solution Developer

We requested feedback from the developer regarding their study participation experience. Their insights are summarized below:

1. Initial functional testing helped the AI model meet the minimum quality requirements.
2. Maintaining the technical defects rate below the thresholds necessitated monthly monitoring over the AI performance and infrastructure, along with prompt resolution of emerging issues.
3. Regular monthly reviews of model performance by an expert radiologist, along with the model leaderboard, encouraged consistent updates and monitoring for data biases.
4. Feedback from radiologists (detailed comments or "agree/disagree" responses) yielded higher-quality hypotheses that enhanced the AI model.
5. Clear functional and diagnostic requirements allow focusing on the system's quality and reliability, rather than specific needs of medical facilities and healthcare practitioners.
